# Effects of Peptide YY on the Hypothalamic-Pituitary-Gonadal Axis in Healthy Men

**DOI:** 10.1210/clinem/dgz103

**Published:** 2019-10-19

**Authors:** Chioma Izzi-Engbeaya, Sophie Jones, Yoshibye Crustna, Pratibha C Machenahalli, Deborah Papadopoulou, Manish Modi, Christos Panayi, Jessica Starikova, Pei Chia Eng, Maria Phylactou, Edouard Mills, Lisa Yang, Risheka Ratnasabapathy, Mark Sykes, Isabella Plumptre, Ben Coumbe, Victoria Wing, Ewa Pacuszka, Paul Bech, James Minnion, George Tharakan, Tricia Tan, Johannes Veldhuis, Ali Abbara, Alexander N Comninos, Waljit S Dhillo

**Affiliations:** 1 Section of Endocrinology and Investigative Medicine, Department of Medicine, Imperial College London, London, UK; 2 Department of Internal Medicine, Mayo Clinic, Minnesota; 3 Department of Endocrinology, Imperial College Healthcare NHS Trust, London, UK

**Keywords:** peptide-YY, luteinizing hormone, follicle stimulating hormone, testosterone, reproduction

## Abstract

**Context:**

Central and peripheral administration of peptide YY (PYY) has potent anorectic effects, and PYY analogs are under development as antiobesity treatments. Recent animal data suggest PYY may also influence the reproductive axis; however the effects of PYY on the human reproductive system are unknown.

**Objective:**

To investigate the effects of PYY administration on the reproductive axis in healthy young men.

**Design:**

Single-blind, randomized, placebo-controlled crossover study.

**Setting:**

Clinical Research Facility, Imperial College Healthcare NHS Trust.

**Participants:**

Eighteen healthy eugonadal men (mean age 24.1 ± 0.9 years, mean body mass index 22.2 ± 0.4 kg/m^2^).

**Intervention:**

Eight-hour intravenous infusion of 0.4 pmol/kg/min PYY_3-36_ or rate-matched vehicle infusion.

**Main Outcome Measures:**

Number of luteinizing hormone (LH) pulses, LH, follicle stimulating hormone (FSH), and testosterone levels.

**Results:**

The number of LH pulses (mean number of LH pulses/8 hours: PYY 4.4 ± 0.3 vs vehicle 4.4 ± 0.4, *P* > .99), LH area under the curve (AUC) (PYY 1503 ± 79 IU.min/L vs vehicle 1574 ± 86 IU.min/L, *P* = .36), FSH AUC (PYY 1158 ± 513 IU.min/L vs vehicle 1199 ± 476 IU.min/L, *P* = .49) and testosterone AUC (PYY 10 485 ± 684 IU.min/L vs vehicle 11 133 ± 803 IU.min/L, *P* = .24) were similar during PYY and vehicle infusions.

**Conclusions:**

Acute intravenous infusion of 0.4 pmol/kg/min PYY does not affect the reproductive axis in healthy men.

Obesity is a major global health problem that increases morbidity (1), mortality (2), and has a significant detrimental impact on healthcare budgets (3). In 2016, 39% of adults and 13% of children worldwide were obese, and more people died as a consequence of obesity than undernutrition (4). Bariatric surgery is the most effective treatment for obesity, but it is not universally available or acceptable to patients, and postprocedure complications limit its use (5). Lifestyle modifications are difficult to maintain outside research settings with extended care required for weight loss maintenance (6). Therefore, medications remain the mainstay of obesity management. An emerging class of antiobesity medication is peptide YY (PYY) analogs, with over 20 patents for this new class of medication listed on the European Patent Register (7).

Physiologically, PYY is predominantly produced by intestinal L cells in response to nutrient ingestion. Resulting rapid elevations in circulating postprandial PYY levels are directly proportional to the size of the ingested meal (8, 9). PYY has a powerful anorectic effect via activation of central Y2 receptors (10) with central and peripheral administration of PYY dose-dependently reducing food intake by up to 25% in rodents (10). In normal weight, overweight, and obese people, administration of PYY potently suppresses appetite and reduces food intake (by ~30%) (10, 11).

Furthermore, there is evidence that PYY may have other effects in addition to weight loss, which are imperative to decipher given the ongoing development of PYY analogs as antiobesity therapies. PYY stimulates the release of luteinizing hormone (LH) and follicle-stimulating hormone (FSH) from isolated prepubertal rat pituitaries (12). Additionally, PYY administration increases LH and FSH levels in adult male rats, an effect that is potentiated by fasting (13). Secondary hypogonadism occurs in up to 40% of obese men, which is associated with higher body weight and increased insulin resistance (14). Therefore, the anorectic effects of PYY, and the potential to stimulate reproductive hormone release, would be advantageous in the treatment of obesity with coexisting hypogonadism.

As there are no reports of the effects of PYY on the reproductive system in humans, we undertook a randomized single-blinded placebo-controlled crossover study to determine the effects of PYY administration on reproductive hormone release in healthy men. Fertility is dependent on absolute reproductive hormone levels as well as LH pulsatility (15). Therefore, we sought to determine the effect of PYY administration on several key parameters of LH secretion, as well as circulating FSH and testosterone levels.

## Materials and Methods

### Study participants

This study was approved by the West London Research Ethics Committee (16/LO/0391) and performed in accordance with the Declaration of Helsinki. Healthy eugonadal men (aged 18–40 years) were recruited via online and print advertisements. Written informed consent was obtained from each participant prior to study enrolment. Exclusion criteria included: body mass index (BMI) <18.5 or >25 kg/m^2^, history of medical and psychological conditions, use of prescription, recreational or investigational drugs within the preceding 2 months, blood donation within 3 months of study participation, ingestion or inhalation of nicotine-containing substances within 3 months, alcoholism, and history of cancer.

### Study visits

Each participant attended 2 study visits, 1 for PYY administration and 1 for vehicle administration. Infusion order was randomized and participants were blinded to the infusion identity. PYY infusions were prepared by dissolving PYY_3-36_ (Bachem, UK) in 1 ml of 0.9% NaCl (Braun, UK) and adding the PYY solution to 49 ml of Gelofusine (Braun, UK). PYY was infused at a rate of 0.4 pmol/kg/min, a dose previously established to be biologically active in humans (9, 16). Vehicle infusions consisted of Gelofusine (Braun, UK), administered at the equivalent rate to the PYY infusion for each participant.

**Figure 1.**
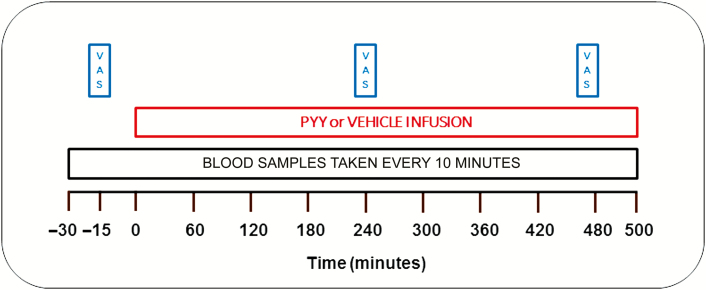
Study visit protocol. After a period of acclimatization following their arrival at the Clinical Research Facility, 18 healthy men (mean age 24.1 ± 0.9 years, mean body mass index 22.2 ± 0.4 kg/m^2^), received an 8-hour infusion of 0.4 pmol/kg/min Peptide YY_3-36_ (PYY) during one study visit and rate-matched vehicle infusion at a second study visit, in random order. Blood samples were taken at ten-minute intervals starting from 30 minutes before the infusion was started (ie, T = –30 minutes). Visual analog scales (VASs) were completed by the participants pre-infusion (T = –15 minutes), mid-infusion (T = 240 minutes) and prior to the end of the infusion (T = 470 minutes).

After an overnight fast starting at 10 pm on the night before each study visit, each participant ate a standardized 200 kcal breakfast (1 pot of Oat So Simple® porridge, Quaker Food Products, UK) at 6 am on the morning of each study visit. The participants arrived at the Clinical Research Facility at 8.15 am on the morning of each study visit. After a period of acclimatization, 2 intravenous cannulae (1 in each arm) were inserted (1 for blood samples and 1 to administer the infusion). Following baseline sampling, PYY or vehicle infusion was started at T = 0 minutes (9 am) and infused until T = 500 minutes. Visual analog scales (VASs, 0–10 cm), used to measure participants’ self-reported nausea, were performed at T = –15 minutes, T = 240 minutes, and T = 470 minutes. Blood samples were taken at 10-minute intervals throughout the study (Fig. 1). Participants were not allowed to eat during the infusion until after T = 480 minutes.

### Biochemical analyses

PYY was measured using an established in-house radioimmunoassay (17). Serum insulin, plasma glucose, serum LH, serum FSH, and serum testosterone were measured in the Clinical Chemistry Laboratory of Imperial College Healthcare NHS Trust on the automated Abbott Architect® platform. Chemiluminescent immunoassays were used to measure serum insulin (intra-assay and interassay coefficient of variation (CV): ≤7%), serum LH (intra-assay and interassay CV: ≤5%), serum FSH (intra-assay and interassay CV: ≤10%), and serum testosterone (intra-assay and interassay CV: ≤8%). Plasma glucose was measured using a colorimetric hexokinase assay (intra-assay and interassay CV: ≤2%).

### Statistical analysis

LH pulsatility was determined using blinded deconvolution analysis (18). Longitudinal nonindependent data were analyzed with generalized estimating equations (GEEs). Paired t-tests were performed on parametric data and Wilcoxon matched pairs sign rank tests were performed on paired nonparametric data. STATA 14.1 (STATACorp, USA) and Prism 8.0.2 (GraphPad, USA) software were used to perform statistical analyses. *P*-values <.05 were considered statistically significant. Data are presented as mean ± standard error of the mean.

## Results

### Participants

Twenty-four healthy men were recruited, with 18 men (mean age 24.1 ± 0.9 years, mean BMI 22.2 ± 0.4 kg/m^2^) completing the study. Four men did not complete the study due to their other commitments and 2 were unable to tolerate the PYY infusion due to nausea. Data from the 18 men who completed the study are included in the analyses below.

### Effects of PYY on LH levels

**Figure 2.**
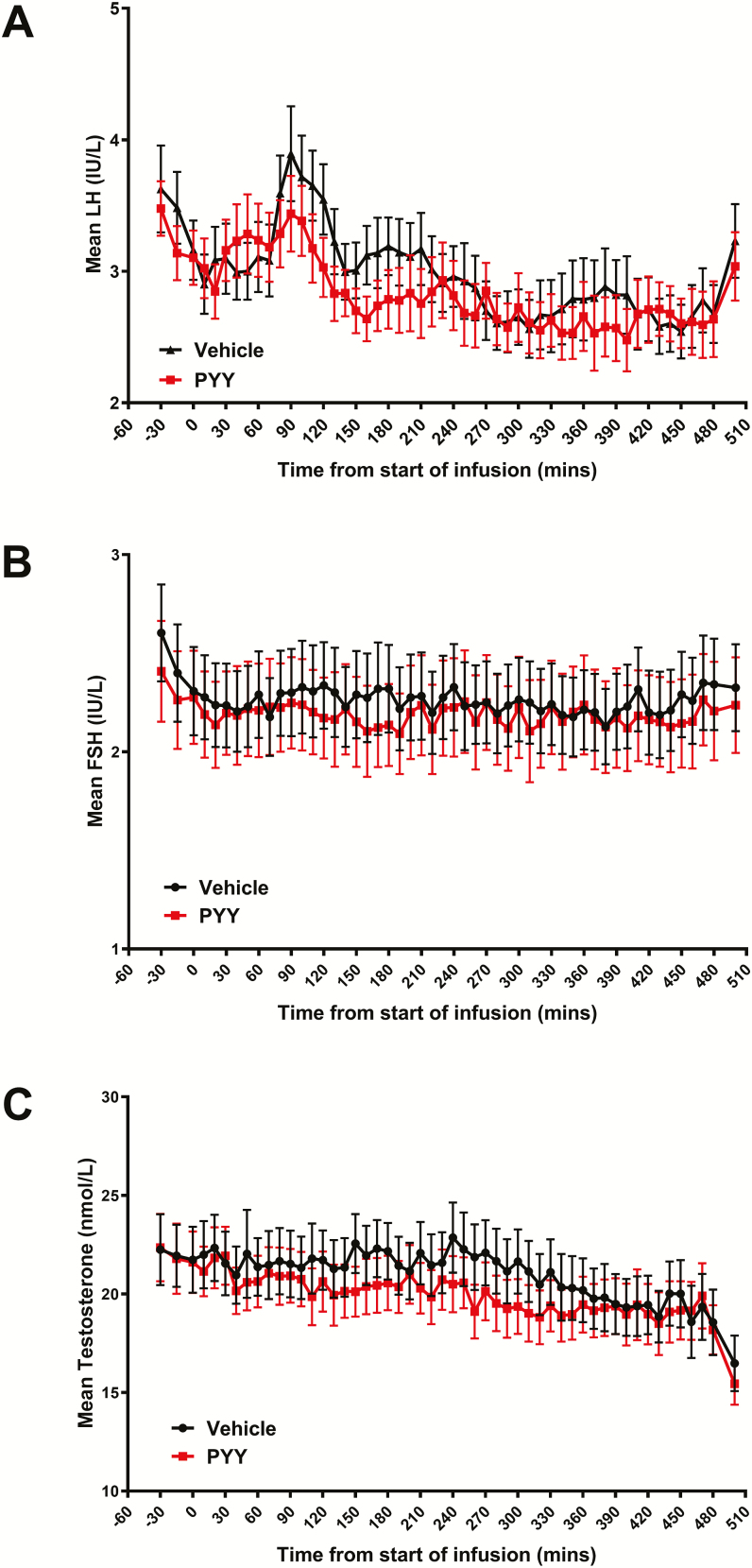
Effects of intravenous peptide YY (PYY) administration (0.4 pmol/kg/min PYY_3-36_ for 8 hours) on reproductive hormone secretion in 18 healthy men. (**A)** Serum luteinizing hormone (LH) levels were similar throughout PYY and rate-matched vehicle infusions (generalized estimating equation (GEE), *P* = .50). (**B)** Serum follicle-stimulating hormone (FSH) levels were similar throughout PYY and rate-matched vehicle infusions (GEE, *P* = .79). (**C)** Serum testosterone levels were similar throughout PYY and rate-matched vehicle infusions (GEE, *P* = .53).

There was no significant difference between serum LH levels during PYY infusion and during vehicle infusion (Fig. 2A). Intravenous PYY infusion did not alter LH pulsatility (mean number of LH pulses/8 hours: PYY 4.4 ± 0.3 vs vehicle 4.4 ± 0.4, *P* > .99). Furthermore, mean LH (PYY 2.8 ± 0.2 IU/L vs vehicle 3.0 ± 0.2 IU/L, *P* = .31) and LH area under the curve (AUC) (PYY 1503 ± 79 IU.min/L vs vehicle 1574 ± 86 IU.min/L, *P* = .36) were not significantly altered by PYY infusion.

### Effects of PYY on FSH levels

Similar to LH, there was no significant difference between serum FSH levels during PYY infusion and during vehicle infusion (Fig. 2B). Additionally, mean FSH (PYY 2.2 ± 0.2 IU/L vs vehicle 2.3 ± 0.2 IU/L, *P* = .48) and FSH AUC (PYY 1158 ± 513 IU.min/L vs vehicle 1199 ± 476 IU.min/L, *P* = .49) did not significantly differ between PYY and vehicle infusion.

### Effects of PYY on testosterone levels

Consistent with the absence of an effect on LH and FSH secretion, intravenous PYY infusion did not alter serum testosterone levels during the 8-hour infusion, and diurnal variation in testosterone levels was unchanged by PYY (Fig. 2C). Similarly, mean testosterone (PYY 19.8 ± 1.3 IU/L vs vehicle 21.1 ± 1.5 IU/L, *P* = .24) and testosterone AUC (PYY 10 485 ± 684 IU.min/L vs vehicle 11 133 ± 803 IU.min/L, *P* = .24) were unaffected by PYY infusion.

### Effects of PYY on nausea and fullness

**Figure 3.**
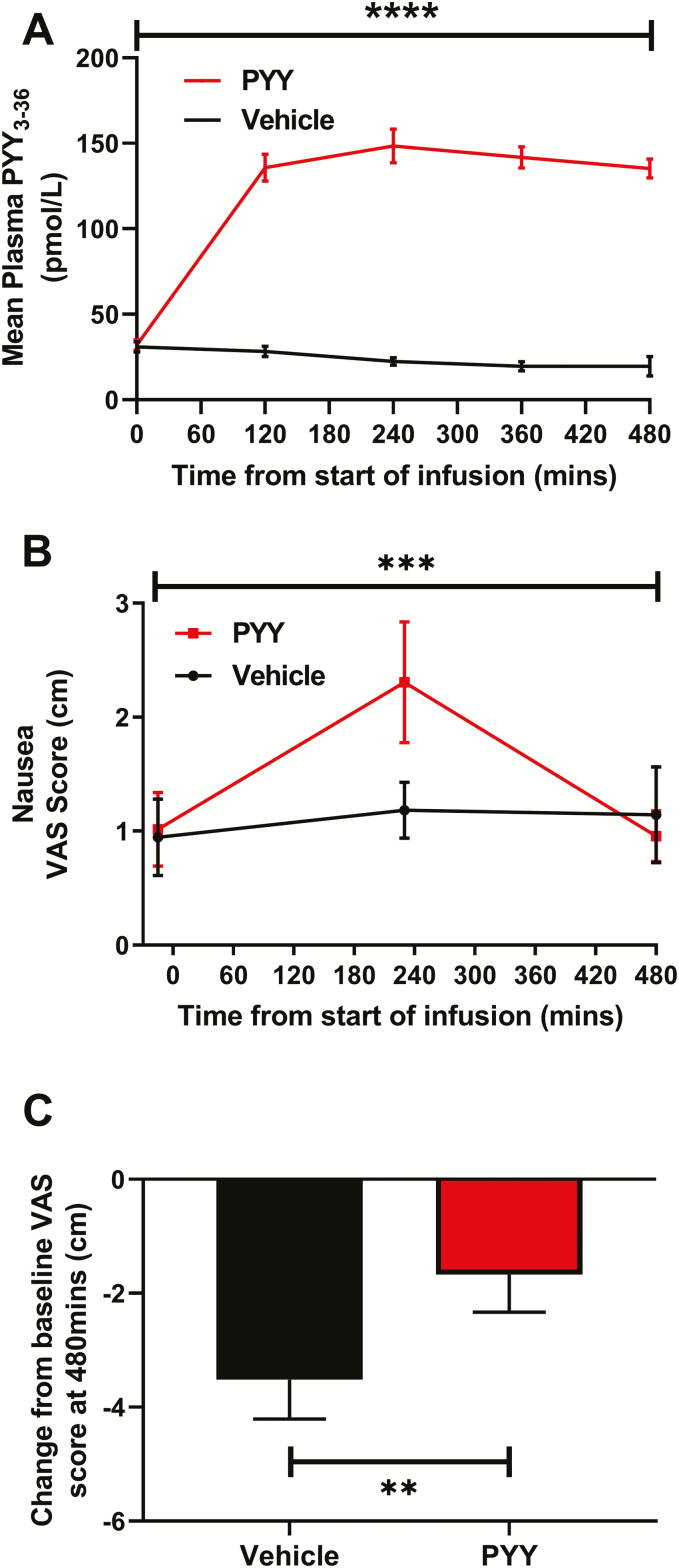
Effects of intravenous peptide YY (PYY) administration (0.4 pmol/kg/min PYY_3-36_ for 8 hours) on plasma PYY levels, nausea and fullness in 18 healthy men. (**A)** PYY infusion resulted in significantly higher plasma PYY levels compared to vehicle infusion (generalized estimating equation (GEE), ****P < .0001). (**B)** Self-reported nausea, measured using a 0–10 cm visual analog scale (VAS), was increased by PYY infusion compared to vehicle infusion (generalized estimating equation (GEE), ***P < .001). **C:** Self-reported fullness, measured using a 0–10 cm visual analog scale (VAS), reduced by a lesser extent at T = 480 minutes during PYY infusion compared with vehicle infusion (Wilcoxon matched-pairs signed rank test, **P = .01).

PYY infusion resulted in significantly higher circulating PYY levels than vehicle infusion (Fig. 3A). PYY is known to cause nausea at biologically active doses (9). Therefore, we assessed the effect of the PYY infusion on nausea. PYY infusion resulted in significantly higher self-reported nausea than vehicle infusion over the 8-hour study period peaking midinfusion (Fig. 3B). However, the absolute nausea levels were low (ie, ~2/10 cm). Additionally, PYY infusion resulted in a smaller reduction in the feeling of fullness at T = 480 minutes compared with vehicle infusion (Fig. 3C).

## Discussion

This is the first study investigating the effects of PYY on the reproductive system in humans. Our study demonstrates that, an 8-hour infusion of 0.4 pmol/kg/min PYY_3-36_ does not alter LH pulsatility in healthy men and does not change circulating levels of LH, FSH, and testosterone. This is in contrast to rodent studies, where PYY administration (centrally or peripherally) had specific effects on reproductive hormone levels depending on pubertal status and route of administration as follows. Incubation of pituitaries from prepubertal rats with PYY results in increased LH and FSH secretion within 60 minutes (12). However, incubation of PYY with hypothalamic fragments from prepubertal and adult rats results in reduced gonadotrophin-releasing hormone (GnRH) secretion (12, 13). Central administration of PYY to prepubertal rats inhibited LH secretion but did not affect FSH secretion (12). In contrast, intracerebroventricular administration of PYY increases LH and FSH in fed and fasted adult male rats, and co-administration of a GnRH antagonist abolishes these effects (13). However, peripheral administration of PYY (via intraperitoneal injection) has no effect on LH secretion but increases FSH secretion in prepubertal male rats (12).

It is possible that no effect on reproductive hormone secretion was detected in response to PYY administration in this study due to the peripheral route of administration. As outlined in the rodent studies above, central administration of PYY stimulates gonadotrophin secretion in adult male rats (13), whereas peripheral administration has been shown to increase FSH (but not LH) in prepubertal male rats (12).

The anorectic effects of PYY are thought to be mediated by agonism of the Y2 receptor (10, 19). However, the effect of PYY on reproductive hormone secretion may not occur via the Y2 receptor. Central administration of a selective Y2 receptor agonist reduces LH and FSH secretion in adult male rats, while administration of a Y2 receptor antagonist increases LH and FSH secretion in adult male rats (20). In contrast, central administration of PYY to adult male rats stimulates LH and FSH secretion (13). In the present study, peripheral PYY administration increased nausea but had no effect on reproductive hormone levels. Therefore, Y2 agonism in healthy men does not modulate reproductive hormone secretion.

Although participants were instructed to fast overnight and eat a standardized breakfast (which was provided to them in advance), the overnight fast and consumption of the standardized breakfast were not monitored. However, this limitation is unlikely to have significantly influenced the results as all participants were closely monitored as fasting throughout the 8-hour infusion and no reproductive hormone changes were evident at any point.

In this present study, PYY administration did not alter LH, FSH, and testosterone levels in healthy (non-obese) men. Further studies are required to determine the effects of PYY on reproductive hormone secretion in obese people, ie, the target population for PYY-based therapeutic agents.

## Conclusions

Although animal data suggest that PYY affects reproductive hormone secretion, our data demonstrate that in humans, acute administration of a biologically active dose of PYY does not have an adverse effect on LH pulsatility and does not alter levels of LH, FSH, and testosterone. This has important clinical and safety implications for the continuing development of PYY analogs for the treatment of obesity.

## Abbreviations

BMI: body mass index
GEE: generalized estimating equation
GnRH: gonadotrophin-releasing hormone
FSH: follicle-stimulating hormone
LH: luteinizing hormone
PYY: peptide YY
VAS: visual analog scale

## Additional Information

***Disclosure Summary:*** The authors have nothing to disclose.

***Data Availability:*** The datasets generated during and/or analyzed during the current study are not publicly available but are available from the corresponding author on reasonable request.

## References

[1] Guh DP, Zhang W, Bansback N, Amarsi Z, Birmingham CL, Anis AH The incidence of co-morbidities related to obesity and overweight: a systematic review and meta-analysis. BMC Public Health. 2009;9:88. 19320986 10.1186/1471-2458-9-88 PMC2667420

[2] Global BMIMC, Di Angelantonio E, Bhupathiraju Sh N, Wormser D, Gao P, Kaptoge S, et al. Body-mass index and all-cause mortality: individual-participant-data meta-analysis of 239 prospective studies in four continents. Lancet. 2016;388(10046):776–786. 27423262 10.1016/S0140-6736(16)30175-1 PMC4995441

[3] Kent S, Green J, Reeves G, et al; Million Women Study collaborators Hospital costs in relation to body-mass index in 1·1 million women in England: a prospective cohort study. Lancet Public Health. 2017;2(5):e214–e222. 29253487 10.1016/S2468-2667(17)30062-2 PMC6196771

[4] Organization WH. Obesity and Overweight Factsheet 2018. https://www.who.int/en/news-room/fact-sheets/detail/obesity-and-overweight. Accessed 25 May 2019.

[5] Puzziferri N, Roshek TB 3rd, Mayo HG, Gallagher R, Belle SH, Livingston EH Long-term follow-up after bariatric surgery: a systematic review. JAMA. 2014;312(9):934–942. 25182102 10.1001/jama.2014.10706 PMC4409000

[6] Middleton KM, Patidar SM, Perri MG The impact of extended care on the long-term maintenance of weight loss: a systematic review and meta-analysis. Obes Rev. 2012;13(6):509–517. 22212682 10.1111/j.1467-789X.2011.00972.x

[7] Office EP. European Patent Register. https://register.epo.org/smartSearch?searchMode=smart&query=peptide+yy. Accessed 25 May 2019.

[8] Gribble FM, Reimann F Enteroendocrine cells: chemosensors in the intestinal epithelium. Annu Rev Physiol. 2016;78:277–299. 26442437 10.1146/annurev-physiol-021115-105439

[9] Degen L, Oesch S, Casanova M, et al Effect of peptide YY3-36 on food intake in humans. Gastroenterology. 2005;129(5):1430–1436. 16285944 10.1053/j.gastro.2005.09.001

[10] Batterham RL, Cowley MA, Small CJ, et al Gut hormone PYY(3-36) physiologically inhibits food intake. Nature. 2002;418(6898):650–654. 12167864 10.1038/nature00887

[11] Batterham RL, Cohen MA, Ellis SM, et al Inhibition of food intake in obese subjects by peptide YY3-36. N Engl J Med. 2003;349(10):941–948. 12954742 10.1056/NEJMoa030204

[12] Fernandez-Fernandez R, Aguilar E, Tena-Sempere M, Pinilla L Effects of polypeptide YY(3-36) upon luteinizing hormone-releasing hormone and gonadotropin secretion in prepubertal rats: in vivo and in vitro studies. Endocrinology. 2005;146(3):1403–1410. 15564330 10.1210/en.2004-0858

[13] Pinilla L, Fernández-Fernández R, Vigo E, et al Stimulatory effect of PYY-(3-36) on gonadotropin secretion is potentiated in fasted rats. Am J Physiol Endocrinol Metab. 2006;290(6):E1162–E1171. 16390861 10.1152/ajpendo.00469.2005

[14] Dandona P, Dhindsa S Update: hypogonadotropic hypogonadism in type 2 diabetes and obesity. J Clin Endocrinol Metab. 2011;96(9):2643–2651. 21896895 10.1210/jc.2010-2724 PMC3167667

[15] Knobil E, Plant TM, Wildt L, Belchetz PE, Marshall G Control of the rhesus monkey menstrual cycle: permissive role of hypothalamic gonadotropin-releasing hormone. Science. 1980;207(4437):1371–1373. 6766566 10.1126/science.6766566

[16] Neary NM, Small CJ, Druce MR, et al Peptide YY3-36 and glucagon-like peptide-17-36 inhibit food intake additively. Endocrinology. 2005;146(12):5120–5127. 16150917 10.1210/en.2005-0237

[17] Tan TM, Salem V, Troke RC, et al Combination of peptide YY3-36 with GLP-1(7-36) amide causes an increase in first-phase insulin secretion after IV glucose. J Clin Endocrinol Metab. 2014;99(11):E2317–E2324. 25144632 10.1210/jc.2014-2143 PMC4258604

[18] Jayasena CN, Abbara A, Veldhuis JD, et al Increasing LH pulsatility in women with hypothalamic amenorrhoea using intravenous infusion of Kisspeptin-54. J Clin Endocrinol Metab. 2014;99(6):E953–E961. 24517142 10.1210/jc.2013-1569 PMC4207927

[19] Abbott CR, Small CJ, Kennedy AR, et al Blockade of the neuropeptide Y Y2 receptor with the specific antagonist BIIE0246 attenuates the effect of endogenous and exogenous peptide YY(3-36) on food intake. Brain Res. 2005;1043(1-2):139–144. 15862527 10.1016/j.brainres.2005.02.065

[20] Pinilla L, Fernández-Fernández R, Roa J, Castellano JM, Tena-Sempere M, Aguilar E Selective role of neuropeptide Y receptor subtype Y2 in the control of gonadotropin secretion in the rat. Am J Physiol Endocrinol Metab. 2007;293(5): E1385–E1392. 17785504 10.1152/ajpendo.00274.2007

